# Nutritional counselling interactions between health workers and caregivers of children under two years: observations at selected child welfare clinics in Ghana

**DOI:** 10.1186/s12913-019-4692-y

**Published:** 2019-11-08

**Authors:** Christiana Nsiah-Asamoah, Kingsley Kwadwo Asare Pereko, Freda Dzifa Intiful

**Affiliations:** 1 Department of Clinical Nutrition and Dietetics, University of Cape Coast, Cape Coast, Ghana; 2 Department of Community Medicine, University of Cape Coast, Cape Coast, Ghana; 3 Department of Dietetics, School of Biomedical and Allied Health Sciences, University of Ghana, Accra, Ghana

**Keywords:** Nutrition Counselling, Health workers, Caregivers, Child welfare clinics

## Abstract

**Background:**

This study evaluated the Health Works (HWs) nutritional counselling skills and information shared with caregivers. This was a cross-sectional study in which an observation checklist was used to examine Growth Monitoring and Promotion (GMP) activities and educational/counselling activities undertaken by health workers (HWs) to communicate nutrition information to caregivers, depending on the ages of the children.

**Methods:**

A total number of 528 counselling interactions between health workers and caregivers in 16 Child welfare Clinics (CWCs) in two rural districts in Ghana were observed. Frequencies were presented for the information that was obtained from each caregiver and those that were provided by the HWs during the nutritional counselling sessions.

**Results:**

About 95.1 and 61.8% of the caregiver-HW interactions involved mothers of children who were less than 6 months of age and those above 6 months respectively. HWs counselled the caregivers on appropriate nutrition for the child. Health talk messages that were shared with caregivers focused mainly on the importance of attending CWCs and vaccination of children and rarely included any teaching materials. In most of the interactions, HWs made of child’s feeding practices the past 1 month; and also did not provide advice on specific issues of IYCF. Nutritional counselling information given for non-breastfeeding children was inadequate and in some cases absent. Little attention was given to the feeding of children with animal products during counselling.

**Conclusion:**

Generally nutritional information given to caregivers who had children above 6 months was inadequate.

## Background

Adherence to recommended infant and young child feeding (IYCF) practices has been identified as a key area that can help tackle undernutrition and ultimately improve child health [1]. In addition, Summers and Bilukha (2018) [2] reported that introducing complementary foods at 6 months of age is the third leading child health intervention in preventing child deaths. As a result of the evidence of the impact of appropriate feeding on child health, the WHO and UNICEF recommend early initiation of breast-feeding within the first hour of birth, exclusive breast-feeding for the first 6 months of life, continued breast-feeding until 2 years of age, and timely introduction of nutritious complementary foods at 6 months of age [3]. Despite these recommendations, global estimates based on analysis of the Multiple Indicator Cluster Survey (MICS), Demographic Health Survey (DHS) and other nationally representative data sources obtained between 2010 and 2016 by UNICEF revealed that about half (48%) of all children aged 6–23 months are not being fed the minimum number of times recommended for their age daily (minimum meal frequency). Again, less than one third (28.2%) are fed a minimal diverse diet comprising at least four out of seven food groups in a day [4]. When both the minimum meal frequency and minimum diet diversity are taken into consideration, only one in 6 (16%) children are receiving a minimally acceptable diet [5].

IYCF counselling was introduced as an integral part of child welfare services following the global challenge of inadequate and poor child feeding practices. In the case of children under 2 years, the need to offer nutritional counselling services especially to their caregivers has received much attention. This stage of life has been described as a critical window of opportunity for ensuring appropriate growth and development through optimal feeding, that reduces the risk of undernutrition and give children the best start in life [6–8]. In addition, children below 2 years are at a higher risk of becoming malnourished and its effect on child’s cognitive development particularly because, it is the stage in life when the body is quickly laying down its fundamental building blocks for brain development and future growth [9]. Therefore, feeding under 2 year olds’ inadequate complementary foods, in addition to poor feeding practices coupled with being affected by frequent episodes of infections during this period are direct risk factors that subject children to malnutrition [10]. If nutritional needs are not met and undernutrition is not addressed at this stage, it leaves long-lasting damages which are largely irreversible [11].

Hence,interventions in the form of nutritional counselling services and health educational talks that are offered to caregivers with children below two years have been recommended as effective strategies that can help improve infant and young child feeding (IYCF) practices, reduce child undernutrition and hence enhance child survival [9, 12, 13]. However, Reinsma et al., (2016) [14] noted that although most of the nutrition counselling in sub-Saharan Africa is primarily conducted by nurses or health volunteers, little is done to develop capacity for nutrition at the professional, organizational, or systemic levels. In addition, Kohli and Chadha (2017) [12] asserted that, studies that focus on assessing nutritional counselling activities and skills of health workers are scarce.

The limited evidence from past studies suggests that HWs may lack specific knowledge on recommended IYCF practices regarding feeding frequencyand portion sizes of complementary foodsthat are given to young children [15]. In addition, findings of previous related studies also indicate that generally, HWs do not have adequate knowledge about the consistency of complementary foods [16] and how to improve the energy/ nutrient density of meals offered to young children [15]. For example, in the study by Chaturvedi et al. [15] in Gujarat, India, only 12% of the HWs were able to correctly advise caregivers on portion sizes of complementary foods, 20% on feeding frequency, 37% on consistency of meal and 30% on improving energy/ nutrient density of complementary foods.

Hence, a study which focuses on investigating the kind of nutritional information and guidelines imparted to caregivers of children under two years is worthwhile considering reports that nutritional interventions that are implemented before two years yield benefits a lifetime, even without the need to be reinforced [17].

While there are a number of studies that have assessed the knowledge levels of HWs on recommended IYCF practices, few have been conducted to evaluate their nutritional counselling activities and skills by observing their practices in their work settings. In Ghana, all the previous studies were conducted in urban areas in the Greater-Accra Region [13, 18] and Brong-Ahafo Region [19]. Also, the findings on the nutritional counselling practices of HWs were based on their own reports or those from mothers who accessed child health services in health facilities. A general observation made in these studies was that, in most cases there were inconsistencies between the responses of mothers and those of HWs regarding HW counselling approaches and nutritional information received by caregivers. The current state of knowledge regarding the nutritional counselling activities and skills of HWs, particularly in rural settings in Ghana, is limited. This study therefore explores and evaluates HWs nutritional counselling skills and information shared with caregivers who access CWCs for GMP services.

## Methods

### Study setting

The study was conducted at + 16) public child welfare clinics (CWCs) located in two rural districts - Kwahu Afram Plains North (KAPND) and Kwahu Afram Plains South (KAPSD) - within the Eastern Region of Ghana. These two districts were purposively selected from the 26 districts in the Eastern Region on the basis of their ranking as the first and second districts with the highest prevalence of underweight in the Region in both 2013 and 2014. In 2013, the prevalence of underweight ranged between 0.02 and 20.85%.

KAPND and KAPSD were ranked as the first and second districts with an underweight prevalence of 20.85 and 18.21% respectively among children under 5 years [District Medium Term Development Plan, 2014–2017]. Similarly in 2014 within a range of 0.4 and 22.0%, KAPND and KAPSD had an underweight prevalence of 22.0 and 16.3% respectively.

### Participants

Using a structured observational counselling checklist, a total number of 528 counselling observations were made in all the 16 CWCs during “weighing days of the 16 CWCs” between August and December, 2018. This study is part of a project that sought to obtain information about household and maternal factors that influence the feeding and nutritional status of children under 2 years. Counselling sessions between HW and mothers were observed. The number of Health worker-caregiver interaction sessions that were observed at a particular CWC facility depended on the number of clients who attended the child ‘weighing’ sessions per clinic day. All eligible mother-child pairs - were sampled, using the consecutive sampling method until the estimated sample size to be selected from each health facility was attained. Out of a total number of 610 mothers who responded to a questionnaire that assessed maternal and household determinants of feeding children, 528 mothers had time and agreed to wait to have an interaction with the HW after the weights of their children had been measured. The remaining 82 mothers indicated that they had no time to wait but had to rush back to the market or the farm immediately after the weights of their children had been measured and recorded. Therefore in this study, respondents’ response and refusal rates were approximately 86.6 and 13.4% respectively. The HWs who were observed at the CWCs were community health nurses (CHNs), community health workers (CHWs), enrolled nurses, disease control officers and midwives.

### Study design and data collection procedures

The study was cross-sectional and employed a qualitative approach to collect data. The investigator with the assistance of two trained field assistants attended the monthly CWC sessions and observed the manner of conducting growth monitoring and promotion activities, with particular emphasis on nutritional counselling interactions between caregivers and health workers at the 16 CWCs. Field notes were taken in a field diary.

An observation checklist was used to examine Growth Monitoring and Promotion (GMP) activities and educational/counselling activities undertaken by health workers (HWs) to communicate nutrition information to caregivers, depending on the ages of the children. The observation checklist was developed on the basis of guidelines by the Ghana Health Service, as specified in the child health records book and WHO child feeding recommendations. Some of the items on the observation checklist were adapted from the studies of Agbozo (2016) [13], Laar (2015) [20] and Gyampoh et al. (2014) [18]. The checklist covered sections on activities conducted before and during weighing of children and growth promotion actions undertaken by HWs after weighing and charting child’s weight. Other items on the checklist evaluated the ability of HWs to record and chart growth curves properly.

The child health records of the child were consulted to assess whether the HWs recorded the monthly weights on the appropriate chart, depending on the sex of the child and to the nearest 0.1 kg. To find out about the growth pattern of the child (whether rising or falling, once or twice, or remaining the same for two consecutive months), the researchers referred back to the growth charts of each child.

The items on the checklist guided observations made by the researcher to appraise nutritional counselling activities of the HWs. The researcher and two other field assistants sat in a non-obstructing position and observed activities of the health workers before, during and after weighing of each child at each of the CWCs. To assess data recording and charting, a child’s growth chart was observed to find out if previous monthly weights were recorded to the nearest 0.1 kg and recorded on the appropriate chart for the sex of the child.

Data on the child’s growth pattern in the last two consecutive months were also collected secondarily from the child growth charts to assess for the proportion of children who had their growth curves rising, falling once, falling in two consecutive months or remained the same for two consecutive months.

Observations were also made of approaches employed by health workers to communicate nutrition information on recommended IYCF practices to caregivers on each health facility’s CWC day. In all the 16 CWCs, information was sought on the topics discussed with caregivers on that CWC’s “weighing day” during the health talk session before weighing of children commenced. The researchers also made observations of the types of teaching materials that were used by HWs during health talks and the type of nutrition counselling information given to caregivers, depending on the age of the child.

After seeking the consent of the HWs and caregivers, the researchers and data collection assistants sat at a non-obstructing position and observed caregivers as they went through the various growth monitoring and promotion activities at the various CWCs, using the developed observation checklist. All health workers who gave their consent to participate in the study and to be observed signed an informed-consent form. In the case of the consenting mothers they either signed or thumb-printed on an informed consent form after the purpose of the study had been explained to them. In order to ensure a high level of voluntary participant involvement, participants were well informed about the purpose and possible benefits that can be derived – improvement in child nutrition and health services provided by HWs –from the study. All participants were assured of anonymity and confidentiality by informing them that no information they provide can be traced back to them in reports, presentations and other forms of dissemination. Ethical approval and clearance for the study was granted by the Dodowa Health Research Centre (DHRC), Institutional Review Board (IRB) of the Ghana Health Service (Reference/Identification: DHRCIRB/04/02/17) and the IRB of the University of Cape Coast (U.C.C) (Reference/Identification: UCCIRB/CHLS/2017/02).

### Data analysis

Frequencies and percentages were presented for the information that was obtained from each caregiver and those that were provided by the health worker during the nutritional counselling sessions. Data on the child’s growth pattern in the past two consecutive months prior to the study, collected secondarily from each child’s growth chart, was presented as frequencies and percentages. The data was summarized showing the proportion of children who had growth curves (patterns) that had risen, fallen once, fallen in two consecutive months, or had faltered (remained the same for two consecutive months).

## Results

The background information of the HWs who were observed at the 16 CWCs is presented in Table 1. A total number of 18 HWs provided nutritional counselling and information to caregivers after weighing of children at the 16 CWCs. It is important to note that in 14 of the CWCs only one HW interacted with all the caregivers on one-on-one basis after their children had been weighed. In two CWCs, which were located at the district capital towns and were also the only referral health facilities, two HWs at these CWCs took turns to interact with caregivers because of the large number of children who accessed the facility for GMP services.

**Table 1:** Background characteristics of Health Workers who were observed at the CWCs

Characteristics	n (%)
Sex	
Male	5 (27.8)
Female	13 (72.2)
Position	
Community Health Nurse	7 (38.8)
Community Health Worker	4 (22.2)
Disease Control/Technical Officer	3 (16.7)
Enrolled nurse	2 (11.1)
Community Mental Health nurse	1 (5.6)
Midwife	1 (5.6)
Length of providing GMP services at CWC/RCH	
< 1 year	1 (5.6)
1–4 years	4 (22.1)
5–8 years	9 (50.0)
9–12 years	3 (16.7)
> 12 years	1 (5.6)
^a^Workshops/Trainings attended	
Vaccine Administration	12 (66.7)
Breastfeeding (communication strategies, management, exclusive)	11 (61.1)
CMAM	10 (55.6)
Complementary feeding	7 (38.9)
No training	2 (11.1)

^a^Workshops/Trainings attended: Some HWs have attended more than one workshop or training programme since they started working as professional HWs

As indicated in Table 1, a higher proportion (38.8%) of the HWs were community health nurses. Most (50%) of the participants had between 5 and 8 years of working experience after their professional training (See Table 1). With respect to participation in any training workshop, more than half of the study participants had participated in training workshops in the area of vaccine administration (66.7%), breastfeeding (61.1%) and Community-based Management of Acute Malnutrition CMAM (55.6%).

### Observation of activities undertaken before and during weighing and recording of Children’s weight on growth chart

Observations for a total number of 528 children aged between 0 and 23 months were made. Table 2 is a summary of findings in the observations undertaken before and during weighing and recording of children’s weight on the growth chart. As indicated in Table 2, none of the CWCs calibrated the weighing scales using known standardized weights before weighing children. In all the 528 (100%) weighing sessions that were observed, HWs ensured that children were undressed and had minimal light clothing before weighing them. The majority of infants (93.6%) whose weights were taken using suspended weighing balances (Salter type) were positioned correctly before their weights were measured.

**Table 2:** Observations before and during Weighing and Recording of Children’s Weight on Growth Chart

Abbreviated checklist items	Frequency (%)
Number of HWs per CWC facility	
1–2	3 (18.8)
3–4	7 (43.8)
5–6	5 (31.2)
> 6	1 (6.2)
Activities done before and during Weighing of Children	
Calibrating the weighing scales using known standardized weights (*n* = 16)	0 (0.0)
Starts by checking zero-setting on the weighing scales before weighing	481 (91.0)
Ensures child undresses and is in minimal clothing before weighing	528 (100.0)
Positions infant correctly on suspended balances (Salter type) (*n* = 218)	218 (100.0)
Growth promotion actions taken by HWs after weighing and charting child’s weight	
Health worker tells caregiver weight of child	271 (51.3)
Health worker explains growth pattern of child using the growth chart	146 (27.7)
Health worker enquires about previous illness (*n* = 131) (only in cases where growth pattern decreased or remained same as compared with previous month)	93 (70.9)
Health worker enquires about feeding (in all cases) (*n* = 528)	214 (40.5)
Health worker praises caregiver (only in cases where growth curve is increasing) (*n* = 397)	211 (53.1)
Procedure for Recording and Charting of weights after referring to child’s growth chart and recorded details of the growth curve (*N* = 528 observations)	
Weights recorded to the nearest 0.1 kg	521 (98.7)
Weight recorded on appropriate chart for sex of child	523 (99.1)
Weight charted properly	528 (100.0)
Weight recorded accurately against appropriate age of child (counts the number of months since birth and check if recorded in appropriate place)	528 (100.0)

### Growth promotion actions taken by HWs after weighing and charting child’s weight

The GMP actions undertaken by HWs after weighing and charting the children’s weights in their child health record booklet are also summarized in Table 2. Out of 528 children, aged between 0 and 23 months, whose weighing was observed by the researchers, the HW informed the caregiver about the weight of the child in only 51.3% of the cases.

As shown in Table 2, it is in the case of only 27.7% of the caregivers that the HW who measured the weight of the child explained the growth pattern of the child, using the growth chart. Health Workers, in some cases (40.5%), inquired from the caregiver whether the child was eating as expected. The results also show that, only a little over half (53.1%) of caregivers whose children’s weight had increased were praised by the HWs.

### Growth pattern of child after referring to child’s growth chart

As indicated in Table 3, out of 528 children whose weighing was observed, the growth curves of the majority (75.2%) were rising - that is, their weights increased as compared with the previous month’s weight. However, the growth pattern of 12.9, 3.9 and 8.0% were found to have respectively fallen once, fallen for two consecutive months and remained the same for two consecutive months.

**Table 3:** Growth pattern of children and procedures for recording and charting of weight

Abbreviated checklist items	Frequency (%)
Growth Pattern of child after referring to child’s growth chart	
Growth pattern: Rising	397 (75.2)
Growth pattern: Falling once	68 (12.9)
Growth pattern: Falling two consecutive months	21 (3.9)
Growth pattern: Falling or same for two consecutive months	42 (8.0)
Growth Pattern of children based on Age Groups	
0–5 months (*N* = 143)	
Growth pattern: Rising	141 (98.6)
Growth pattern: Falling once	0 (0.0)
Growth pattern: Falling two consecutive months	0 (0.0)
Growth pattern: Falling or same for two consecutive months	2 (1.4)
6–23 months (*N* = 385)	256 (66.5)
Growth pattern: Rising	68 (17.7)
Growth pattern: Falling once	21 (5.4)
Growth pattern: Falling two consecutive months	40 (10.4)
Growth pattern: Falling or same for two consecutive months	
Procedure for Recording and Charting of weights after referring to child’s growth chart and recorded details of the growth curve (*N* = 528 observations)	
Weights recorded to the nearest 0.1 kg	521 (98.7)
Weight recorded on appropriate chart for sex of child	523 (99.1)
Weight charted properly	528 (100.0)

All the cases of growth patterns which either fell once or for two consecutive months occurred among children aged between 6 and 23 months. In addition, out of 42 cases of falling growth patterns (where the weights of the child remained the same for two consecutive months) 40(95.2%) were children aged between 6 and 23 months. It is recommended and expected that caregivers of these affected children will be counselled and supported to improve the nutritional status and growth rate of their children. However, a general observation made was that HWs who came into contact with such children usually inquired by asking the caregiver why the weight of the child was not increasing or had decreased. In other cases, the HW asked the caregiver whether the child had been sick in the past weeks. In the case of previously sick children as shown in Table 3, some HWs advised caregivers on the need to give additional meals after illness and foods that are liked best by the child during such periods of sickness and recovery. Other HWs also advised caregivers who had reported that their children had been sick in the past weeks to breastfeed more often and give soft and easily digestible foods. None of the HWs provided information on why it was necessary to employ these feeding strategies during periods of sickness and recovery of young children. Some HWs also asked the caregiver whether the child was eating well as expected. None of the HWs asked for any detailed information about specific foods that were given to the child or the feeding practices of caregivers during periods of sickness.

### Observations of nutrition Counselling services given by health workers to caregivers

#### Scope of Counselling messages shared with caregivers

Generally, on a typical CWC/weighing day, the first activity undertaken by HWs was to conduct a health talk to all caregivers by employing a mass/group discussion approach. In all the 16 facilities, the health talks were usually led and facilitated by a Community Health Nurse or midwife.

Five (5) facilities had developed a planned monthly health talk programme indicating topics that would be discussed with caregivers during health talks. Generally, in all these five facilities, topics for the health education sessions were similar. The topics that were displayed on the wall at the premises of the CWCs included *importance of attendance at CWCs and immunization of children, importance of personal and environmental hygiene, prevention of malaria, importance of family planning, importance of Antenatal and Postnatal Care (ANC and PNC), importance of exclusive breastfeeding (EBF), prevention of home accidents, mental health issues, management of fever, prevention of HIV,* and *hand washing*. Other topics that were not common to all the five facilities included importance of complementary feeding, importance of hospital delivery, importance of sleeping under insecticide treated bed nets, importance of sex education and prevention of cholera.

#### Approaches employed in counselling caregivers

In most cases, the leaders/facilitators of the health talk usually shared their messages by employing a didactic one-way approach, which was followed-up with questions by the HW; and caregivers were given an opportunity to answer and share their views. In the 16 facilities, the duration for delivering health talks ranged between 20 and 30 min. With regard to the approach employed by HWs to convey nutrition information to caregivers, in all the 16 CWC facilities, nutritional education activities were conducted for the entire group of caregiver (in the form of giving a health talk), at the beginning of the GMP session, before HWs proceeded with weighing of children. One-on-one counselling approach between a HW and caregivers was employed after weighing each child. In all the 16 CWCs one-on-one counselling was done in an open area, usually under a tree where a table and two chairs had been placed for a HW and caregiver to interact. There were no special counselling rooms to ensure privacy between HWs and their clients in any of the 16 CWCs that were visited. In addition, generally the one-on-one interactions between HWs and caregivers were more of a one-way approach during which the HW did most of the talking. In all the interaction sessions that were observed, HWs did not negotiate with caregivers and hardly presented options available to improve the child’s situation. There was no case where a HW discussed with a caregiver on matters regarding options caregivers could afford or do to help improve the nutritional status of the child. In all the observations that were made, there was no case where a HW and caregiver come to any agreement on feeding and care practices that must be employed to improve the child’s nutritional status. Since no agreements were made, no information was documented in the child health record booklet to guide any follow-up process during next visits to the CWC.

As shown in Table 4, only 6 out of the 16 health facilities studied used teaching aids/visual aids (TAs/VAs) during health talks and one-to-one counselling with caregivers.

**Table 4:** Observations of topics discussed with care givers and use of teaching materials

Abbreviated checklist items	Frequency (%)
Topics discussed with caregivers on “weighing” day (*N* = 16)	
Importance of attending CWCs and vaccination of children	5 (31.3)
Importance of exclusive breastfeeding	4 (25.0)
Importance of personal and environmental hygiene/hand washing	2 (12.5)
Complementary feeding and foods to introduce to child	2 (12.5)
Mental health education	2 (12.5)
Prevention of malaria and sleeping under insecticide treated bed net	1 (6.2)
Use of Teaching Materials in health facility (*n* = 16)	
Yes	6 (37.5)
Teaching Materials used by HWs during Health Talks (*n* = 6)	
Child Health Record Booklet, developed by GHS	4 (66.7)
Counselling Cards on IYCF for Community Health Workers, developed by UNICEF	2 (33.3)

One of the teaching aids used by HWs was the Child Health Record Booklet developed by the Ghana Health Service (GHS), which has pictorial illustrations on foods for complementary feeding, good positioning and attachment for breastfeeding, foods to be fed to a healthy child and feeding frequencies per day for different age groups (pages 12 and 13). The other teaching material used by the HWs was the counselling cards on IYCF for Community Health Workers, developed by UNICEF. This counselling card presents brightly coloured illustrations which depict key infant and young child feeding concepts that can be shared with caregivers.

#### Nutritional counselling of caregivers with infants aged between 0 and 5 months old

In the current study, with respect to caregivers with infants aged between 0 and 5 months, the majority (95.1%) were counselled to practice exclusive breastfeeding (see Table 5). Again, 88.8% of them were apprised of the importance of breastfeeding, and 58.7% of them were counselled and encouraged to practice good hygiene, including before breastfeeding. However, only 19.6% were taught and received guidelines on breastfeeding techniques. None of the caregivers with children below 6 months were educated on how to express breast milk.

**Table 5:** Observations of Nutrition Counselling Services Given by Health Workers to Caregivers

Abbreviated checklist items	Frequency (%)
Nutrition Counselling Information Given	
Counselling of Caregivers with infants 0–5 months old (*n* = 143)	
Encourages exclusive breastfeeding	136 (95.1)
Explains the importance of breastfeeding	127 (88.8)
Teaches breastfeeding technique	28 (19.6)
Advises feeding on demand	72 (50.3)
Educates on expressing breast milk	0 (0.0)
Counsels and encourages good hygiene practices (*including before breastfeeding)*	84 (58.7)
Encourages caregiver to ask questions and answers them	21 (14.7)
Enquiries made after weighing of children 6–23 months old (*n* = 385)	
Asked about feeding frequency	107 (27.8)
Probed about portion size	0 (0.0)
Enquired about consistency of food being fed to the child.	0 (0.0)
Took feeding history	73 (18.9)
Asked mothers about any iron rich foods being given to the child	0 (0.0)
Asked mothers about any vitamin A rich foods being given to the child	0 (0.0)
Sought information about the morbidity history of the child	124 (32.2)
Nutritional Counselling of caregivers with children 6–23 months old (n = 385)	
Encourages and counsels on complementary feeding	238 (61.8)
Encourages continued breastfeeding to 2 years and beyond in addition to complementary feeding	147 (38.2)
HW generally encourages frequent feeding in a day	202 (52.4)
Provided specific advice on feeding frequency appropriate for child’s age	94 (24.4)
Provided advice on portion sizes to be fed to child	0 (0)
Provided advice on food consistency	0 (0)
Promoted diet diversity in their discussion with caregivers	76 (19.7)
Advised caregivers about giving iron- rich foods to children	163 (42.3)
Counsels and encourages hygiene practices especially when breastfeeding, cooking and feeding child	186 (48.3)
Counselling on Feeding Child During Illness (*n* = 93)	
Need to give additional meals after illness	23 (24.7)
Need to give small frequent feeds during illness	19 (20.4)
Give foods that the child likes	63 (67.7)
Continue to breastfeed	42 (45.2)
Give soft and easily digestible foods	31 (33.3)
HW indicates number of times of feeding meals (*n* = 385)	
1–2 times for breastfed infants 6–8 months *(n = 126)*	23 (18.3)
3–4 times for breastfed children 9–23 months *(n = 245)*	71 (28.9)
4 times for non-breastfed children 6–23 months *(n = 14)*	0 (0.0)
For non-breastfed children, HW advises mother to give at least 2 milk feedings in a day in addition to complementary foods *(n = 14)*	0 (0.0)
HW counsels caregiver to generally feed a variety of foods *(n = 385)*	197 (51.2)
HW counsels caregiver to specifically feed *(n = 385)*	
Cereals (foods prepared from rice, millet, maize etc)	238 (61.8)
Roots and tubers (foods prepared from yam, cassava, plantain etc)	194 (50.3)
Legumes and nuts (beans, cowpea, groundnut etc)	173 (44.9)
Flesh foods (meat, poultry, fish and liver/organ meats)	136 (35.3)
Eggs	98 (25.4)
Dairy foods	76 (19.7)
Vitamin-A rich fruits and vegetables (dark green vegetables, pawpaw, water melon, mangoes, palm nut)	183 (47.5)
Other fruits and vegetables (*mentions examples of locally grown or available fruits and vegetables*)	83 (21.6)
Encourages mother to ask questions and answers them	47 (12.2)

#### Nutritional counselling of caregivers with young children 6–23 months old

As shown in Table 5, only 61.8% of caregivers with children aged between 6 and 23 months were encouraged and counselled on complementary feeding. A little above half (52.4%) of the caregivers were generally advised to frequently feed their children. However, only 94 (46.5%) out of 202 received specific guidelines on the number of meals in addition to snacks, that should be given to a child in a day, again demonstrating the broad nature of the counselling information given by HWs to caregivers.

Regarding the number of times meals should be fed to children daily, only 18.3% of caregivers with children aged between 6 and 8 months were counselled to feed their children on 1–2 meals, in addition to snacks and breast milk. Again, only 28.9% of caregivers with children aged between 9 and 23 months were advised to feed their children on 3–4 meals, in addition to snacks during the day.

There were 14 non-breastfed children who were less than 12 months; but their mothers reported that these infants stopped breastfeeding on their own within one to two months after they started complementary feeding. Specific nutritional information on feeding frequency and the importance of milk-feeding for the non-breastfeeding child was absent in all the 14 caregiver-health worker interactions that were observed. No HW advised any of the 14 caregivers with a non-breastfeeding child to give meals 4–5 times and offer 1–2 snacks as well as milk and milk products daily. Nutritional counselling information provided to caregivers of non-breastfeeding children were not specific but generally centred on broad information, such as feeding child with a wide variety of foods, preparing child’s meal separately and feeding the child with favourite foods.

A high proportion (51.2%) of caregivers with children aged between 6 and 23 months were counselled on the need to feed their children with a variety of foods. Regarding advice given on feeding the child with specific foods, a larger majority (61.8%) of caregivers were counselled on the need to give cereals (foods such as maize porridge, rice, millet and “*banku*”, prepared from fermented corn dough). During the one-to-one interaction between HWs and caregiver, less than half (47.5%) of the caregivers were specifically advised to give Vitamin-A rich fruits and vegetables to their children. Again, 44.9 and 50.3% of the caregivers were specifically advised to give legumes and nuts and roots and tubers respectively to their children.

For all the 528 observations that were made, in approximately 87.1%, HWs did not give any opportunity to their clients to ask questions after the counselling sessions.

## Discussion

Globally, growth monitoring and promotion (GMP) is one of the first health services provided for infants and young children in the early stages of their lives; and it is believed to prevent and manage malnutrition conditions [5, 12].Observations made regarding activities of HWs prior to the commencement of the weighing sessions revealed that no weight measuring scale was calibrated in all of the 16 CWCs. However, as with any measurement instrument, weighing instruments should be calibrated regularly to ensure that they are measuring accurately and consistently using certified calibrated or known weights [21]. A worrying observation made during the 4 weeks period of observing activities at CWCs is that the weighing scales used to measure weights of children in all of the 16 CWC facilities were not calibrated. Calibration of weighing equipment is necessary because scales became increasingly less precise with continuous usage and exertion of weights on them [21]. In addition, to ensure accurate measurements, it has been recommended that weighing scales in health facilities have to be tested or calibrated more frequently, particularly if they are moved around often [22], as in the case of all the weighing scales that were used in the CWC facilities that were observed. The implication of using non-calibrated weighing equipment is the tendency to have inaccurate and inconsistent body weight measurements which can have a negative impact on patient care [22]. Among paediatric populations, non-compliance in obtaining accurate weights can result in delays in establishing treatment and discharge plans for such sick children [23]. The finding in this study is similar to that of a related study undertaken in Norway which reported that only 3 out of 27 (11.1%) child health clinics had good routine procedures with regard to how weighing scales were tested and calibrated [24]. Although the researchers did not find out why the instruments were not calibrated before they were used, one reason that can be attributed to this practice is the likelihood that these HWs have not been educated on the importance of calibrating weighing instruments before their usage and trained on how to carry out the process. Another possible reason could be the unavailability of reference or *standard* known *weights* to *calibrate* weighing instruments. Again, it is likely that HWs ignore the importance of calibrating weighing instruments because of the excuse of not having enough time to spend on such procedures mainly because of the many clients that access the health facility which may result in a work overload on HWs as reported in a related study in Limpopo Province, South Africa [25].

The finding in the current study with respect to GMP actions undertaken by HWs after weighing and charting the children’s weights in their child health record booklet corroborates with a similar study conducted in some CWC facilities in Accra, Ghana, where approximately 40% of caregivers were informed about the weight of their children, and half of them were given nutrition counselling [18].With regard to explaining the growth pattern of a child to a caregiver, using the growth chart; HWs not interpreting the growth pattern of the children to their caregivers could result in some parents consulting other caregivers for explanation or trying to interpret the growth curve on their own. A comparable finding was reported in a related study in Kenya, where only 37.9% of caregivers indicated that the HW explained the growth pattern of their children to them. On the other hand, a higher proportion (54.2%) indicated that they tried interpreting the growth pattern of their children on their own, while others (7.9%) consulted a fellow mother for interpretation [26]. Similarly, in another study undertaken in South Africa, it was observed that the majority of nurses also did not provide feedback on the children to their caregivers, after weighing and recording the weights on the growth chart [27]. It is important to note that providing feedback to caregivers ensures their active participation and interest in dealing with problems associated with the growth of their children [28]. In the present study, the researchers did not find out why HWs were not giving any feedback to caregivers after weighing their children. However, some inferences can be made on the basis of observations that were made. Not providing feedback to caregivers could be attributed to reasons such as the unawareness of the importance of giving feedback to caregivers, nurses work overload possibly as a result of the inadequate number of trained personnel to attend to the numerous children. As a result of the high workload, HWs are likely not to attach much importance to the need to give feedback to caregivers in order to avoid spending much time per client.

The IYCF Counselling package recommends that HWs providing GMP service should inform caregivers about their children’s body weight, and then ask them to interpret the growth curves of their children, on their own. This is because the growth chart is considered a monitoring and educational tool that enables both HWs and caregivers to visualize the growth trajectory of children and informs them of the appropriate actions to take [29].Weighing of children in the presence of caregivers also provides a better opportunity for communication with caregivers about the status of the child’s growth, by making growth visible. It also results in better targeting of preventive activities to detect and halt falling growth patterns before it progresses to the state of undernutrition [30]. Explanations given to a caregiver on a child’s growth pattern, followed by individual-tailored counselling, based on the growth status of the child, is highly recommended in GMP activities [31]. Bilal et al. (2014) [29] added that the recorded information on the child should be interpreted and communicated among HWs and caregivers of the respective children from as early as birth until the children reach at least 5 years of age. This is recommended as one of the positive GMP counselling skills, perhaps because it ensures that caregivers are given the opportunity to fully participate in monitoring the growth of their children and to readily accept suggestions given for improving upon the growth of their children.

Generally, health workers disregarded taking detailed information on the feeding history of children, which entails finding out about what the child was being fed with, the frequency of feeding and the manner in which the child was fed. Similar to this finding, Chaturvedi et al’s [15]. study in Gujarat, India indicated that the inability of HWs to inquire about the feeding history of children and provide need-based advice was persistent during counselling sessions with caregivers. The practice of not taking detailed information on the feeding history of children could be attributed probably to HWs not knowing about its importance as a means to help caregivers to improve upon the dietary intakes of their children. Another possible reason could be excuses that are likely to be given by HWs of their inability to spend much time per caregiver to obtain detailed information because of their work overload and the few number of HWs posted to each facility to offer GMP services to many children.

With respect to praising caregivers when offering GMP services, according to *The Community Infant and Young Child Feeding (IYCF) Counselling Package* developed by UNICEF for HWs, if a HW observes that there are no apparent difficulties in the feeding and growth of the child, the HW conducting the GMP service should praise the mother (or caregiver) and focus on providing information needed for the next stage of the child’s development. However, only a little over half (53.1%) of caregivers whose children’s weight had increased were praised by the HWs. Likewise, Maramagi et al.’s [32] study in Uganda reported that the performance of HWs was poor in respect of praising caregivers for their good childcare practices which were evident in the growth of their children. In the study by Maramagi et al. (2004) [32], only 43% of caregivers who engaged in appropriate childcare practices were praised by HWs. Similarly, Gyampoh et al. (2014) [18] in their study in Accra, Ghana, reported that only 2.6% of caregivers whose children were experiencing an improvement in growth were praised for good practices by HWs. Clearly, these findings suggest that HWs do not attach much importance to the need to praise caregivers to motivate and spur them on to continue their good child care practices, when the growth of their children progresses as expected.

The topics which guide health talks organized for caregivers who access CWC services fell within the objectives of GMP which aims at achieving the following results: increasing adequate diet intake by children, improving child care practices among caregivers, increasing the need for health care services among caregivers, improving infection prevention and control of diseases [33].

With regard to approaches employed in counselling caregivers, in similar studies, health talks emphasizing key nutrition and IYCF messages were delivered at the beginning of a day’s activities in child health facilities [13, 34]. According to Agbozo, (2016) [13] counselling services to caregivers could be provided collectively for a group of caregivers or on an individual basis. There is evidence that both approaches (individualized and mass), when employed during counselling of clients, could result in improvements in the nutritional knowledge of caregivers and in children’s feeding and nutritional status [35]. In addition, Bhutta et al. (2013) [10] asserted that a combination of both individual and group counselling approaches results in better impacts on infant nutritional and health outcomes.

The findings revealed that no HW shared any information on expressing and storing breastmilk to enable an infant to consume it in the absence of the mother. An explanation that can be given to the finding that no mother with a child below 6 months was educated on how to express breast milk is that most of the caregivers were farmers, had their own jobs and could go to work with their babies and breastfeed them, unlike other caregivers who have to resume work after a maternity leave of about 3 months. Most salary-earning mothers who resume work after a maternity leave may have to resort to expressing breast milk, to enable the baby to have adequate breast milk while at work. This may not be the situation with this population in which most of the women are self-employed, engaged in farming or are traders. Therefore, HWs in this district are not likely to attach very much importance to educating mothers on expressing and storing breast milk.

The finding that a little above half of the caregivers with children above 6 months were counselled on recommended child feeding practices is supported by similar studies in which less than half of caregivers who attended child health facilities received advice on complementary feeding from HWs [18, 35]. The findings presented in this and other studies suggest, therefore, that to a large extent a high proportion of caregivers are not counselled on appropriate IYCF practices when they visit health facilities for GMP services. Therefore, this defeats one of the objectives of GMP which aims at improving child care practices among caregivers by promoting adequate dietary intakes of children.

A comparison between counselling information given to caregivers with babies below 6 months and those with children above 6 months shows that whereas a higher proportion (95.1%) of caregivers with infants below 6 months received nutritional counselling information, in the case of caregivers with children aged between 6 and 23 months, only 61.8% were counselled. In a similar study, whereas 65% of caregivers reported receiving nutritional counselling on exclusive breastfeeding of babies, only 7% were counselled on complementary feeding [36]. Likewise, the findings of this study are supported by those of Samuel et al. (2016) [16] who found that, whereas the majority (79%) of CHWs reported counselling caregivers on practical tips on breastfeeding, less than half (46%) counselled mothers on complementary feeding practices. Clearly, these findings suggest that generally, less emphasis is placed by HWs on providing caregivers with the needed information on appropriate complementary feeding practices than on exclusive breastfeeding. Related to the findings on feeding frequency counselling information are the results from a study in India, in which only 19% of interactions between caregivers and HWs included counselling on feeding frequency appropriate for children’s ages [15]. A general observation made in this current study was that, for children who were above 1 year, HWs rarely mentioned the importance of continued breastfeeding until the child was 2 years, or even beyond.

With respect to feeding non-breastfeeding children 6–24 months of age, according to WHO’s guiding principles, such children should be provided with meals 4–5 times each day. In addition, nutritious snacks (such as pieces of fruit or bread with nut paste) should be offered 1–2 times per day, as desired. In the case of non-breastfed children aged 6–23 months, they should also be given milk or milk products two or more times a day to ensure that their calcium needs are met, since they are not breastfeeding [3]. However, in this study, no HW advised any of the 14 caregivers with a non-breastfeeding child to give meals 4–5 times and offer 1–2 snacks as well as milk and milk products daily. Nutritional counselling information provided to caregivers of non-breastfeeding children were not specific but generally centered on broad information, such as feeding child with a wide variety of foods, preparing child’s meal separately and feeding the child with favourite foods.

The findings show that much emphasizes was laid by the HW on giving specific foods mainly grown on a large scale locally and that were readily available in the districts. This practice of HWs is commendable, since WHO recommends that *mothers* of young *children s*hould be encouraged as much as possible to identify and use indigenous home-grown foodstuffs, which would help ensure that *local foods* are utilized in homes [37]. Similarly, in a related study conducted among caregivers attending health facilities to access GMP services in nine (9) districts in the Brong Ahafo Region of Ghana, HWs also encouraged caregivers to use locally available and affordable foods within their districts [19].

In this study, with regard to mentioning specific foods that can be given to children, eggs and dairy products were not mentioned often by HWs. Similarly, the study by Gyampoh et al. (2014) [18] also reported that eggs were not mentioned to any of the caregivers in the study, whereas dairy foods were cited to only 0.9% of mothers. Egg is an animal protein food source which contains all the nine essential amino acids required to support growth in children. Compared with other animal food products, eggs are more affordable than meat and fish. Considering that the study was conducted in rural districts in which most of the inhabitants are poor farmers, one would have thought that much more emphasis would have been laid on consuming eggs to promote the growth of children in these districts.

With regard to the feeding of children during periods of sickness, in only a few of the one-to-one interactions, 24.7 and 20.4%, were caregivers counselled on the need to give additional meals after illness and frequently give small portion sizes of food during illness respectively. Such advice was only observed in some interactions between HWs and caregivers of children (*n* = 93) whose weight had decreased or remained the same as the previous month’s weight. A comparable finding was reported in a study conducted in India, in which as low as 18 and 6% of HWs advised caregivers to give small frequent feeds during illness and give an additional meal after illness respectively [38]. Another observation made in this study was that caregivers were not often advised on the need to continue breastfeeding when the child was sick. It was in only 45.2% of the caregiver-HW interactions that emphasis was laid on the need to continue breastfeeding when the child was unwell. This contrasts with the study of Parikh & Sharma [38] which was conducted in Gujarat, India, in which only 6% of HW stressed on the need for continued breastfeeding in situations where children were sick.

The finding that HWs generally do not give caregivers an opportunity to ask questions after the nutritional counselling session corroborates with other related studies which also reported that HWs did not usually invite questions from caregivers or allow caregivers to ask questions after counselling interactions [18, 39]. In the study by Gyampoh et al. (2014) [18], it was indicated that only 4.9% of caregivers were given an opportunity to ask questions after they had received nutrition counselling from the HW. According to Kourkouta and Papathanasiou (2014) [40], one principle of effective interpersonal communication is to ask questions to assess the extent to which the caregiver understood the information that had been given by the HW. Both the Nutrition Assessment, Counselling and Support (NACS) User’s Guide, developed by the Food and Nutrition Technical Assistance III Project (FANTA) 2016, and UNICEF’s Training of Trainers Manual for Counselling on Maternal, Infant and Young Child Nutrition (2011) recommend that, to make the nutrition counselling sessions more educative, interactive and effective, HWs should encourage clients to ask questions. Inviting questions from caregivers is a learned communication skill that encourages two-way communication between the HW and the caregiver, and can also serve as a reinforcement strategy to improve compliance to IYCF guidelines given by HWs. Questions that are left unasked may hinder the way caregivers accept and act on suggestions given on appropriate IYCF practices by HWs [41]. A major factor that could discourage HWs from inviting questions from their clients is the tendency to believe that it wastes their time and drags their work activities [32]. In addition, other factors such as the number of HWs assigned to provide the counselling services and the availability and time commitments of HWs could affect the provision of counselling messages by HWs and their uptake by caregivers [42, 43].

There are some limitations associated with this study. First, the number of CWCs (16) that were studied by conducting observations to assess caregiver-health worker interactions during growth monitoring and promotion (GMP) activities is not representative of the total number of child-health facilities in Ghana. Since the study was restricted to only caregivers with children who visited CWCs for GMP services, the observations made does not include what pertains with GMP services that are provided during community outreach programmes and in Community-based Health Planning Services (CHPS) zones. As such the findings cannot be generalized to all settings where GMP services are provided.

Second, the presence of the researchers and field assistants may have also had some influence on the health workers who were carrying out GMP activities. Therefore the observation of skills and practices might not have depicted the entire real situation related to provision of GMP services, and may have only captured what happened on days that observations were conducted, as compared to the reality with respect to the daily discharge of GMP duties by HWs. Nevertheless, the study of 16 randomly selected CWCs and the use of the observation technique can be regarded as precursors of reliable and trustable information rather than interviewing or administering a questionnaire to HWs who are likely to evaluate or report their own activities more positively.

## Conclusions

The present study was aimed at evaluating nutritional counselling interactions between HWs and caregivers of children under 2 years. However, it became evident that the interaction between the caregivers and HWs can be described more as a presentation of information to caregivers and was rarely a two-way form of communication. HWs did not discuss and confer with caregivers on available options that could be adopted in order to improve the child’s nutritional status.

The study also highlighted that health workers rarely used any teaching aids (visual aids) during their interactions with caregivers. For children aged between 0 and 5 months, the majority of HWs counselled mothers on the need and importance of exclusive breastfeeding. However, one aspect of infant nutrition that was not highlighted by any of the HWs during their interactions with the caregivers was how to express and store breastmilk.

In the case of children aged between 6 and 23 months, the majority of HWs did not make any enquiries about feeding frequencies, consistency of foods, iron and vitamin - A - rich foods that were fed to the children. In counselling caregivers on specific foods to give, whereas more emphasis was laid on giving cereals, roots, tubers, legumes and nuts, less attention was paid to giving dairy products and eggs. In addition, although the HWs advised caregivers to frequently feed their children on green-leafy food items because of their high iron content, their messages did not emphasize the need to add food items, such as oranges, that enhance the absorption of non-heme iron present in plant food sources. There were gaps in the nutritional information that was shared with caregivers on recommended feeding practices for children during illness and the recovery period. Nutritional information given to caregivers of non-breastfeeding children was inadequate and sometimes absent, as no HW advised them to give at least four meals, in addition to two milk feedings daily.

One limitation associated with this study is the issue on volunteer *bias* (or *self-selection bias*) on the part of the mothers, which should be expected in a health facility- based study. In this study the question is whether the mothers who willingly agreed to remain to interact with the HW were different from those who were in a hurry to return back to their farms or the market. However, since 86.6% of the mothers willingly consented to participate in the study, the 13.4% who declined may not affect the samples’ representativeness in this study.

## Recommendations

On the basis of the findings from this study, it is evident that health workers at CWCs should be encouraged to consider openly praising mothers who adhere to recommended IYCF practices, resulting in promoting the growth of their children. Furthermore, with the consent of such mothers, HWs can use their children as examples worth emulating, for other mothers to also strive to improve their child feeding practices. Mothers, including HWs, who are able to exclusively breastfeed their children can also be given an opportunity to share their experiences with other mothers during CWCs.

Obtaining information on the nutrition of children should be given priority by health workers. HWs must take detailed information on the feeding histories of children, providing specific advice on the basis of the individual nutritional needs of children, e.g. non-breastfeeding children. District Nutrition and Public Health officers should be supported and encouraged to pay regular visits to child-health facilities to supervise and monitor the activities of HWs working there as demonstrated in a study by Avula et al. (2011) [44] in India, and also to identify inappropriate GMP practices and contradictory nutrition information that may be shared with caregivers.

One suggestion for further study is to conduct further qualitative studies that will include interviewing HWs in order to understand what they know on child nutrition, why they do what they do, why they do not do what is recommended of the protocol during the provision of GMP services. Another suggestion for further study is to employ more rigorous data collection methods, such as video-recording of HW activities and interactions with caregivers, compared with the use of checklists, which could give a better picture of the issues pertaining to providing healthcare services at child-health facilities in Ghana. Another suggestion for further study is to assess whether the organization of nutrition education programmes for HWs can improve upon their knowledge levels and GMP services offered to children. An additional suggestion is to find out whether nutritional information given to caregivers by HWs influences the feeding practices and nutritional status of their children.

## Data Availability

The data records supporting the information stated in this article regarding the observations that were made using a checklist is available from the corresponding author on reasonable request, without breaching participant confidentiality.

## Abbreviations

ANC: Antenatal Clinic
CHNs: Community Health Nurses
CHPS: Communty-Based Health Planning and Service
CHWs: Community Health Workers
CMAM: Community-Based Management of Acute Malnutrition
CWCs: Child welfare Clinics
DHRC: Dodowa Health Research Centre
DHS: Demographic and Health Survey
EBF: Exclusive Breastfeeding
FANTA: Food and Nutrition Technical Assistance
GHS: Ghana Health Service
GMP: Growth Monitoring and Promotion
HIV: Human Immunodeficiency Virus
HW (s): Health Worker (s)
IRB: Institutional Review Board
IYCF: Infant and Young Child Feeding
KAPND: Kwahu Afram Plains North
KAPSD: Kwahu Afram Plains South
MICS: Multiple Indicator Cluster Survey
NACS: Nutritional Assessment Counselling and Support
PNC: Postnatal Clinic
TAs: Teaching Aids
UNICEF: United Nations Children’s Fund
Vas: Visual Aids
WHO: World Health Organisation
